# Blood Vessels as a Key Mediator for Ethanol Toxicity: Implication for Neuronal Damage

**DOI:** 10.3390/life12111882

**Published:** 2022-11-14

**Authors:** Kei Nakayama, Hiroshi Hasegawa

**Affiliations:** Laboratory of Hygienic Sciences, Kobe Pharmaceutical University, 4-19-1 Motoyamakita-machi, Higashinada-ku, Kobe 658-8558, Japan

**Keywords:** ethanol, brain, blood vessel, angiogenesis, alcoholism

## Abstract

Excessive intake of ethanol is associated with severe brain dysfunction, and the subsequent neurological and behavioral abnormalities are well-established social risks. Many research studies have addressed how ethanol induces neurological toxicity. However, the underlying mechanisms with which ethanol induces neurological toxicity are still obscure, perhaps due to the variety and complexity of these mechanisms. Epithelial cells are in direct contact with blood and can thus mediate ethanol neurotoxicity. Ethanol activates the endothelial cells of blood vessels, as well as lymphatic vessels, in a concentration-dependent manner. Among various signaling mediators, nitric oxide plays important roles in response to ethanol. Endothelial and inducible nitric oxide synthases (eNOS and iNOS) are upregulated and activated by ethanol and enhance neuroinflammation. On the other hand, angiogenesis and blood vessel remodeling are both affected by ethanol intake, altering blood supply and releasing angiocrine factors to regulate neuronal functions. Thus, ethanol directly acts on endothelial cells, yet the molecular target(s) on endothelial cells remain unknown. Previous studies on neurons and glial cells have validated the potential contribution of membrane lipids and some specific proteins as ethanol targets, which may also be the case in endothelial cells. Future studies, based on current knowledge, will allow for a greater understanding of the contribution and underlying mechanisms of endothelial cells in ethanol-induced neurological toxicity, protecting neurological health against ethanol toxicity.

## 1. Introduction: Ethanol Effect on the Nervous System

Historically, ethanol has been the most commonly used drug in the world, and “alcohol” mostly means ethanol in our daily life. Ethanol has various beneficial and harmful effects. For instance, light alcohol intake exerts transient, euphoric, and stress-relieving effects, whereas habitual alcohol use can be addictive and increases the risk of neurobehavioral diseases, inflammation disorders, and bacterial/viral infections [1,2,3,4,5].

Among the diverse toxic effects of ethanol, brain toxicity is especially emphasized because the brain is very susceptible to ethanol exposure, leading in many cases to addiction and the development of behavioral abnormalities. Ethanol affects brain development and functions at all life stages. During infancy, ethanol intake by the mother causes a series of developmental disorders, known as fetal alcohol spectrum disorders. Alcohol drinking at the very early stages of pregnancy increases the risk of miscarriage [6]. In contrast, ethanol consumption at mid to late gestation stages causes tissue dysmorphogenesis, especially in the face and brain [7,8,9]. Excessive ethanol consumption during the third trimester can lead to brain developmental abnormalities, including neuronal cell death and synaptogenic defects, resulting in neuronal plasticity deficit [10,11]. In juveniles, when higher order nervous system functions develop, ethanol intake has been shown to dramatically increase the risks of alcohol use disorders, anxiety-like behavior, impulsivity, and higher risk-taking [12,13,14,15,16]. For these effects of ethanol on the high order brain functions, the susceptibility of the prefrontal cortex (PFC) to ethanol is critically important. Ethanol acutely depresses PFC neuronal activity [17], and chronic binge ethanol drinking disrupts PFC maturation and long-term functions, including decision-making processes, behavioral inhibition, and working memory [18,19,20,21]. However, the histological features and molecular mechanisms governing the higher susceptibility of the PFC to ethanol remain unclear.

In adults, excessive ethanol consumption leads to reversible or permanent changes in brain structures and cognitive functions, although tolerance to ethanol toxicity largely depends on the individual. According to the data obtained from the National Library of Medicine [22], a 1 g/kg body weight (b.w.) dose of ethanol generally results in blood levels of 100–150 mg/dL in most adults. Blood ethanol concentration between 150 and 300 mg/dL causes apparent pathological symptoms in most people. In contrast, the metabolizing machinery is not fully developed in young children; therefore, 50–75 mg/dL of ethanol might be enough to induce significantly adverse effects. Ethanol in blood easily penetrates the blood vessel wall and enters the inside of the brain, regardless of the existence of the blood–brain barrier (BBB), directly affecting neurons and glial cells. Ethanol can also have some effects on the blood vessels themselves. However, few studies have analyzed the effect of ethanol on brain blood vessels.

After oral consumption, ethanol is absorbed through stomach and intestine and transferred to the liver. The liver has high metabolizing activity toward ethanol and generates acetaldehyde. Acetaldehyde is highly toxic to the human body and a major cause of retinal damage in alcoholism. It easily penetrates the BBB, similarly to ethanol, and exerts various effects on the brain, which are different from those induced by ethanol [23]. Some aspects of ethanol toxicity in the brain can be explained by acetaldehyde. For example, acetaldehyde is responsible for the activation of dopaminergic neurons after ethanol consumption [24,25,26]. Notably, acetaldehyde is more chemically reactive than ethanol, being attributable to the carcinogenic effects and epigenetic changes of genomic DNA [27,28]. Because ethanol itself has minimal effect on genomic DNA, the increased risks of cancer as a result of binge alcohol intake largely depend on acetaldehyde [29]. In contrast, metabolism to acetaldehyde attenuates the action of ethanol itself. Acetaldehyde is less potent in inducing hypothermia than ethanol, and metabolism of ethanol into acetaldehyde attenuates the hypothermic effect after ethanol intake [30]. Acetic acid, the final metabolite of ethanol, has been suggested to have some effects of ethanol in neuronal toxicity, although the exact contribution of this molecule is still obscure and remains to be elucidated [31].

In this paper, we summarize recent findings regarding the role of blood vessels in ethanol toxicity in the brain and other tissues, and we discuss their importance in ethanol-induced neurological disorders. Understanding ethanol toxicity in blood vessels is bound to open a new window into considering blood vessels as potential therapeutic or preventative targets for alcoholism-related disorders.

## 2. Effect on Ethanol on Blood Vessel Endothelial Cells

### 2.1. Molecular Target of Ethanol

**Figure 1:**
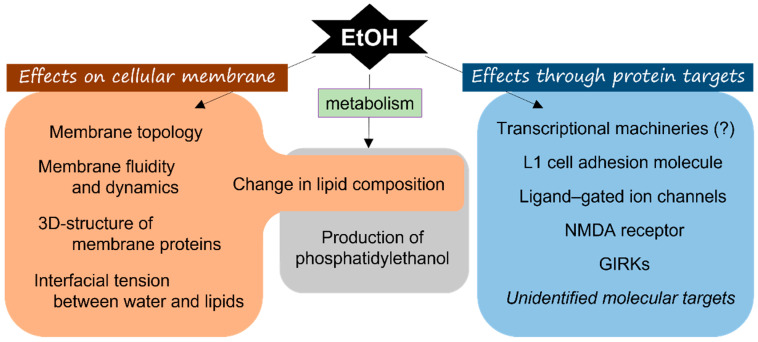
Summary of molecular targets of ethanol in mammals. Ethanol exerts its toxic effects through multiple mechanisms and molecular targets, including modulation of membrane properties and cellular proteins. It also indirectly acts after metabolized to another molecule, such as acetaldehyde, acetic acid, and phosphatidylethanol.

To understand the mechanisms of ethanol-induced toxicity, it is critically important to identify and characterize direct molecular targets of ethanol. However, these molecular targets remain obscure, largely due to difficulties in molecular and pharmacological analyses of this small natural molecule (the molecular weight of ethanol is 46). We summarize current knowledge of this issue in this section (Figure 1).

#### 2.1.1. Direct Action of Ethanol on Membrane Lipids

Classically, membrane lipids are considered the primary target of ethanol. It has been known that chronic ethanol treatment changes the biophysical and biochemical properties of cellular membranes [32]. Ethanol increases the fluidity of all biological membranes, including the plasma membrane [33]. Studies using NMR indicated that ethanol binds to the lipid–water interface of membrane bilayers [34]. A computational simulation study supported this finding by showing that ethanol interacts with the lipid bilayer and causes a slight increase in the surface area per lipid and decrease in the lamellar d-spacing of the bilayer [35]. It also exerts a slight but consistent effect on the membrane dynamics. Especially, ethanol reduces the tension at the membrane–water interface and lateral pressure profile to the membrane–water interface [36]. An in vitro study has further implicated the direct effect of ethanol on synthetic lipid bilayers, suggesting the damaging effect of ethanol on the cellular membrane [37]. Ethanol alters the membrane–water interface by allowing water molecule encroachment into the lipid bilayers, which may alter the conformational space of transmembrane proteins, including ion channels [38,39]. Besides the direct effect of ethanol on membrane lipids, ethanol can be metabolized and incorporated in the membrane lipid, such as phosphatidylethanolamine, sphingomyelin, and platelet-activating factor, which could alter membrane topology and activate intracellular signaling pathways [40,41,42]. Thus, ethanol seems to directly influence the characteristics of the cellular membrane. On the other hand, ethanol is metabolized to phosphatidylethanol, which is not present in an alcohol-free condition, by a transphosphatidylation reaction with phosphatidic acid [43,44]. Phosphatidylethanol increases the secretion of VEGF through the scavenger receptor CD36- and LIMPII analogue-1 (CLA-1)-mediated activation of protein kinase C and p44/42 mitogen-activated protein kinase pathways [45]. Thus, ethanol-metabolized lipids can regulate endothelial cell proliferation through other mechanisms than the regulation of membrane topology and properties.

In addition to the direct effect of ethanol on cellular membrane lipids, ethanol can affect lipid integrity through the production of ROS. ROS and ROS-induced lipid peroxidation are highly reactive to cellular membranes, inducing changes in their properties, such as fluidity [46,47,48]. Ethanol-metabolites in hepatocytes trigger ROS production during both acute and chronic alcoholism [49,50]. In turn, ROS activates the NF-kB pathway to regulate transcription of various genes, including those involved in angiogenesis and blood vessel remodeling [51]. Thus, it is possible that both direct and indirect pathways play distinct roles in regulating membrane lipids.

#### 2.1.2. “Receptor” Proteins of Ethanol

As stated above, ethanol affects the properties of cellular membrane bilayers. However, its direct action on membrane lipids cannot explain all pharmacological effects of ethanol, especially under chronically administered conditions. Recent studies have clarified various proteins as potential “receptors” for ethanol. In *Escherichia coli*, ethanol has been shown to directly act on the ribosome and RNA polymerase, and this interaction is involved in the subsequent ethanol tolerance of bacteria [52]. Although the direct effect of ethanol on transcriptional and translational machineries has not been identified in mammals, this interaction may partly participate in the onset and progression of alcoholic liver disease [53]. In mammals, ethanol has been reported to bind directly and inhibit the adhesive function of L1 cell adhesion molecule, which contributes to the pathogenesis of fetal alcohol spectrum disorders [54,55]. Ethanol interferes with the intramolecular binding of immunoglobulin-like domain 1 and 4 [55]. It has also been revealed that ethanol modulates the activity of ligand-gated ion channels, including GABA_A_, glycine, and ionotropic glutamate receptors [56,57]. Primary binding sites in ion channels for ethanol have been examined with crystal structural analysis using the prokaryote pentametric ligand-gated ion channel. This analysis demonstrated that there was a potential ethanol-binding site on the transmembrane cavity among the channel subunits [58]. This cavity is conserved in human ethanol-sensitive glycine and GABA_A_ receptors [59]. The resulting functional effect of ethanol on channel activity is contradictory and enigmatic, probably because of the differences of administered doses of ethanol [60]. Ethanol also interacts with the NMDA receptor; however, the structural features of this interaction have not been shown. Mutational analysis indicated that the transmembrane domains 3 and 4 are important for the inhibitory action of ethanol on the NMDA receptor [61,62]. Large conductance voltage- and Ca^2+^-gated K^+^ channels (BK channels) are also ethanol targets, and they are believed to be involved in the development of alcohol tolerance and dependence. Ethanol binds to the slo1 cytosolic Ca^2+^-sensing tail domain of BK channels only in the presence of Ca^2+^ [63]. Ethanol directly associates with and activates G protein–gated inwardly rectifying K^+^ (GIRK) channels. The binding site for ethanol on GIRK channels is located in the cytoplasmic cavity formed by the GIRK tetramer [64,65]. Thus, ethanol-binding sites appear diverged among various receptor proteins. One of the potential consensus structural features of ethanol-binding sites has been proposed, which may contribute to the identification of novel ethanol-binding proteins [66].

Interestingly, ethanol shares its binding site on ion channels with small volatile anesthetic molecules, such as barbiturate, enflurane, and isoflurane, whose chemical characters are significantly different from ethanol [67]. Βromoform and propofol can also bind to the ethanol-binding site on glycine and GABA_A_ receptors [59]. Although toluene, a small organic molecule with anesthetic effect, inhibits the NMDA receptor, its binding site is different from that of ethanol [62]. Thus, molecular size is primarily important for the recognition of ethanol/anesthetics by the ethanol-binding proteins, but some chemical characteristics are also important. Further studies are expected to contribute to the development of small and effective anesthetic molecules without significant toxic side effects, such as dependency.

The ethanol-binding proteins stated above are mainly expressed in neurons and glial cells, and there is currently no knowledge of membrane ethanol-binding proteins on endothelial cells. However, some studies have reported the potentially direct action of volatile isoflurane on endothelial cells [68,69,70]. This may indicate the existence of an ethanol “receptor” on endothelial cells, which should be characterized in future studies.

### 2.2. Effect of Ethanol on Endothelial Functions

Endothelial cells, which form a monolayer lining all blood vessels, play crucial roles not only in maintaining blood vessels but also in regulating vascular functions and immune responses during inflammation and infection. These cells are constantly in direct contact with blood at their apical surfaces, and thus they are responsible for protecting tissues from harmful and toxic compounds in blood. Consequently, endothelial cells are susceptible to these harmful and toxic effects. Ethanol is one of those toxic chemicals, which is absorbed in the stomach and intestines and is subsequently transported to all tissues, including the brain, by blood [71]. A previous epidemiological study in Japan revealed that habitual heavy alcohol consumption (46 g/day) increases the risk of endothelial dysfunction, as indicated by branchial artery flow-mediated dilation (FMD) [72]. In vitro, ethanol exposure at physiologically relevant concentrations also damages the endothelial barrier without affecting cell viability in cultured human umbilical vein endothelial cells (HUVECs) and bovine pulmonary artery endothelial cells [73]. Furthermore, ethanol increases the permeability of various types of endothelial cell monolayers via disruption of junctional protein integrity through activation of multiple mechanisms [73,74,75,76,77].

### 2.3. Ethanol Toxicity through the Impairment of the Blood–Brain Barrier

**Figure 2:**
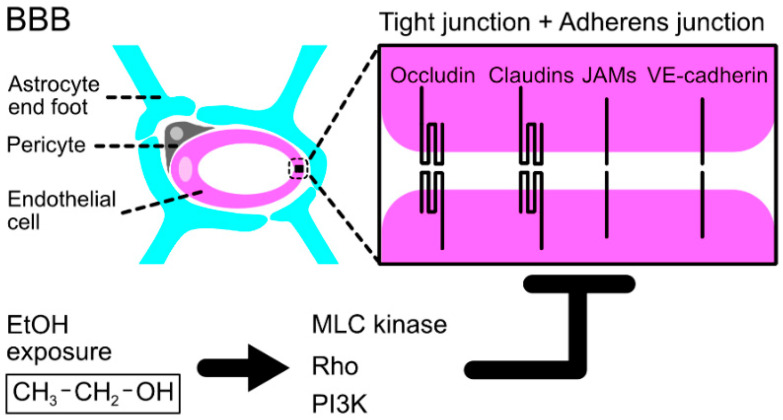
Ethanol exposure affects the integrity of the BBB. The BBB is composed of the vascular endothelial cell, pericyte, and astrocyte to form a tight seal of blood vessels. Ethanol disrupts the integrity of the BBB through the regulation of MLC kinase, small GTPase Rho, and phosphatidylinositol 3-kinase (PI3K).

Ethanol intake impairs the BBB, a specific microvascular structure in the brain, which is responsible for protecting vulnerable neurons from toxic blood components. The BBB is mainly composed of tight-sealed blood vessel endothelial cells linked with the tight junction and adherens junction, astrocytic foot processes, pericytes, and extracellular matrix (ECM) proteins [78,79] (Figure 2). The structures of junctional machinery of endothelial cell, astrocytic processes, and pericytes, as well as the production of ECM proteins at the BBB, are dynamically regulated, enabling the precise movement control of ions, molecules, and leukocytes between blood and parenchyma. Long-term exposure to ethanol leads to the loss of BBB structural integrity [80]. Disruption of epithelial junctions by ethanol is not limited to the BBB and has also been reported in tumor blood vessels, a process which is mediated by actin cytoskeleton changes and unregulated internalization of VE-cadherin, promoting the metastasis of breast cancer cells [73]. In bovine brain microvascular endothelial cells, exposure to ethanol activates myosin light chain (MLC) kinase, the activated MLC kinase phosphorylates MLC, and junctional proteins exacerbate BBB disintegration [81]. This toxic effect of ethanol on the BBB could also be mediated by ethanol-metabolite acetaldehyde and/or increased generation of reactive oxygen species (ROS). The generated ROS affects the integrity and properties of tight/adherens junctions via the regulation of RhoA small GTPase, phosphatidylinositol 3-kinase, and phosphokinase B [82].

In healthy conditions, the BBB function is controlled by various endogenous molecular mediators. Because BBB integrity protects the nervous system from various harmful intrinsic and extrinsic stimuli, tight regulation of the BBB function is critical for maintaining optimal brain health. Therefore, disintegration of the BBB could result in the development of central nervous system (CNS) disorders. Increased BBB permeability is an initial event and central feature of multiple sclerosis (MS) [83,84,85]. BBB dysfunction is also observed in ischemic strokes, in which leukocytes infiltrate into the lesion site through leaky blood vessels and induce pathological inflammation after the stroke onset [86,87]. Epileptic patients also demonstrate BBB leakage; however, the precise role of BBB dysfunction in the pathogenesis of epilepsy remains obscure [88,89,90]. Leaky BBB is implicated as an early biomarker for Alzheimer’s disease [91,92]. Considering the tight relationship between BBB integrity and neurological disorders, ethanol-induced BBB may enhance CNS vulnerability to environmental changes during daily life [93].

In addition to blood vessel endothelial cells, lymphatic endothelial cells are also affected by ethanol. Herrera et al. suggested that MAP kinase p38 plays a crucial role in the ethanol-induced increase in lymphatic endothelial permeability, although this process is not accompanied by expressional changes of tight and adherens junction proteins [94]. The relationship between high number of lymphatic endothelial cells and CNS disorders, such as AD, Parkinson’s disease, and MS, has been implicated [95,96,97,98,99,100]. Thus, the contribution of lymphatic vessel disintegration in ethanol-induced neurological dysfunction should also be examined.

### 2.4. Ethanol Toxicity through Nitric Oxide Production

Nitric oxide (NO) production has been especially emphasized as a mediator of ethanol toxicity. NO is a versatile gas molecule produced by three nitric oxide synthase (NOS) subtypes: neuronal (nNOS), endothelial (eNOS), and inducible (iNOS) subtypes. NO demonstrates both beneficial and detrimental effects on the whole body (reviewed in [101]), and its best-known function is the endothelium-dependent vasodilatation [102]. In addition, NO also plays important roles in neurotransmission and immune cell responses in the brain [103,104,105]. Clinically, NO production is reported to be associated with neurological disorders, such as Alzheimer’s disease, dementia, Parkinson’s disease, ischemia, and migraine [106,107,108].

#### 2.4.1. Roles of NO in Ethanol Toxicity in the Nervous System

NO production has been shown to occur in various brain regions after repetitive ethanol administration in rodent models [109,110]. NO has been known to be involved in various neuronal functions. For instance, nNOS-knockout mice showed reduced ethanol-induced hyper locomotor activity and conditioned place preference, suggesting that nNOS plays a significant role in ethanol-induced behavioral sensitization and conditional response [111]. Alternatively, iNOS, which is generally involved in the upregulated production of cytokines in the brain, is induced and activated by ethanol. Ethanol induces the expression of iNOS through the activation of nuclear factor kappa B (NF-κB) in cultured astrocytes [112]. Moreover, ethanol increased iNOS expression and inhibited the contraction of isolated colon muscles, which were reversed by pretreatment with S-methylisothiourea, a specific inhibitor of iNOS [113]. A recent study has indicated that iNOS mediates the production of inflammatory cytokines after ethanol administration in the kidney [114]. In contrast, the functional roles of iNOS in the ethanol-induced CNS toxicity remain obscure. Contrary to the positive effect of ethanol on iNOS expression and activity stated above, ethanol inhibits the upregulation of iNOS induced by a combinational treatment of inflammatory cytokines, interleukin-1β, tumor necrosis factor-α, and interferon-γ in the C6 glioma cell line [115]. Acute ethanol exposure to astrocytes was found to enhance TNF-α- or interleukin-1β-induced upregulation of iNOS at 50 mM, as opposed to being suppressed at 200 mM in A172 human glioma cell line [116]. This indicates that ethanol has multiple pharmacological targets with different sensitivity. Recently, a study conducted by Chen et al. also indicated that the regulation of NOS proteins is concentration-dependent: low-level ethanol activates eNOS, whereas chronic higher level of ethanol intake induces and activates iNOS. The authors hypothesized that low-level eNOS-generated NO enhances BBB permeability and clearance of waste metabolites, such as amyloid proteins, whereas high-level iNOS-provided NO is responsible for the oxidative damage to neuronal cells [117]. Thus, regulated NO production in an ethanol concentration-dependent manner has diverse physiological functions. The contribution of regulated NO production, especially in endothelial cells, as well as in microglia and astrocytes, to ethanol-induced neurological disorders should be examined in detail in future studies.

#### 2.4.2. Contribution of eNOS to Action of Ethanol on Endothelial Cells

As with the case of iNOS, the effect of ethanol on eNOS expression and activity also depends on the concentration of ethanol and the cellular context of the microenvironment. Treatment of bovine aortic endothelial cells with moderate level (0.1%) of ethanol increased eNOS and NO production [118]. Similarly, treatment with 0.04–0.16% ethanol increased the expression of eNOS and NO production in porcine pulmonary artery endothelial cells, mediated by the phosphatidylinositol 3-kinase pathway [119]. In vivo, ethanol administration to rats (36% of caloric intake) for six weeks increased eNOS expression in the aortic vascular wall [120]. In contrast, higher daily ethanol consumption (blood alcohol levels > 29 mM) for six weeks decreased eNOS expression, increased endothelial cell-derived vasoconstricting prostanoid, and sensitized the responses of mesenteric bed to phenylephrine [121]. However, the exact mechanism of how ethanol regulates expression of eNOS is totally unknown. The expression of eNOS is regulated by histone acetylation and methylation [122], and histone modification is controlled by ethanol (Reviewed in [123]), suggesting a possibility that ethanol controls the expression of eNOS through an epigenetic mechanism.

In addition to mRNA expression, ethanol directly regulates the enzymatic activity of eNOS. Low-to-moderate levels of ethanol (20 mM) increase eNOS activity in HUVECs in a phosphatidylinositol 3-kinase and Akt-dependent manner [124]. It has been previously shown that eNOS activity is controlled by its binding to some proteins. For example, ethanol administration regulates the direct binding of eNOS to heat shock protein-90, caveoilin-1, and calmodulin in porcine pulmonary artery endothelial cells or rat primary hepatocytes [119,125]. Hendrickson et al. reported that ethanol enhanced eNOS activity through the heterotrimeric Gi protein, although it remains obscure how ethanol affects the activity of Gi protein [126].

### 2.5. The Effects of Ethanol on Angiogenesis/Blood Vessel Remodeling

**Figure 3:**
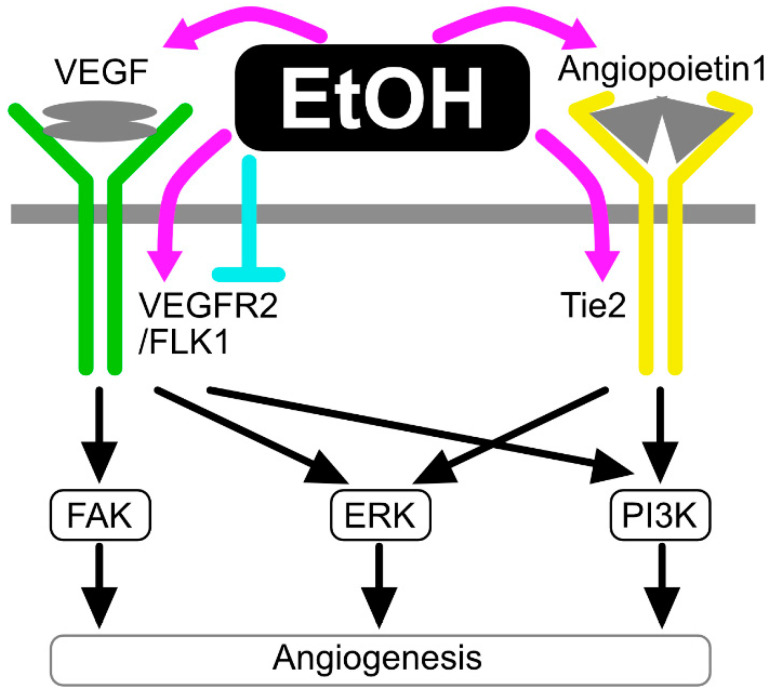
Targets of ethanol in angiogenic signaling. Ethanol controls angiogenesis by modulating VEGF and angiopoietin signaling pathways.

Angiogenesis is associated with neurological disorders. Abnormal angiogenesis is involved in the pathogenesis of Moyamoya disease, which is often accompanied by cognitive and developmental delays or disability [127]. Aberrant angiogenesis and remodeling of brain microvessels is associated with disorganization of the BBB, neuroinflammation, and dysfunction of the neurovascular unit, resulting in neurological deficits [128,129]. In contrast, angiogenesis has curative roles in the recovery from ischemic stroke and depression [130,131]. Thus, maintenance of vascular structure is crucial for normal brain functions. Ethanol stimulates the proliferation and migration of HUVECs (1–100 mM for 24 h) [132]. This angiogenic effect of ethanol has been implicated in cancer progression, especially in breast cancer. Ethanol itself exerts only a faint effect on angiogenesis but significantly enhances estradiol-induced angiogenesis in breast cancer. Preconditioned media from ethanol- and estradiol-treated TG-1 murine mammary cancer cell line increased migration and tubular formation of murine endothelial cell line SVEC4-10 [133]. The increased angiogenesis is accompanied by the enhanced expression of vascular endothelial growth factor (VEGF) in TG-1 cells, suggesting that ethanol stimulates autonomic angiogenic signaling in these cells [133] (Figure 3). In the mouse xenograft model of E0771, a mammary adenocarcinoma cell line, increased angiogenesis and tumor progression were observed after administration of 2% *v*/*v* ethanol for two weeks [134]. In melanoma, chronic alcohol intake also stimulates tumor angiogenesis and progression of tumor growth via the enhanced expression of VEGF, suggesting that the increased angiogenesis induced by ethanol is not limited to a specific type of cancer [135]. It will be interesting to examine whether the synergistic effect of ethanol and other steroid hormones on angiogenesis is involved in tumorigenesis and tumor progression. For instance, estradiol plays important roles in brain development [136], as well as in the protection from neurological diseases in adults [137]. Therefore, it is essential to investigate the synergistic effect of estradiol and ethanol on brain development and functions.

However, literature has also reported the opposite effect of ethanol on angiogenesis. For example, single administration of ethanol at 1.4–1.7 g/kg b.w. was found to reduce angiogenesis in wounded skin [138], perhaps due to the decreased expression of VEGFR2 [139]. Preconditioning of mice with excess dose of ethanol (5 g/kg b.w./day) inhibited angiogenesis after myocardium injury, whereas preconditioning with a low dose (0.5 g/kg b.w./day) was shown to enhance angiogenesis [140]. Thus, the regulation of expression and activity of VEGF signaling is also cell- and context-dependent.

The angiogenic activity of ethanol is reported to be mediated by the upregulation of Notch receptor 1 and 4 mRNAs. The increased expression of Notch 1 and 4 results in the enhancement of Notch signaling, which upregulates the expression of angiopoietin-1 and its receptor Tie2 [132] (Figure 3). The same group demonstrated that ethanol treatment activated VEGF receptor Flk-1, a receptor that is also mediated by Notch signaling in human coronary artery endothelial cells [141]. Activation of Notch signaling is notable among neurological diseases. For example, moderate levels of ethanol administration can inhibit neuronal cell death by serum amyloid via Notch-dependent and -independent pathways [142]. Other than Notch, ethanol is implicated to activate the PI3K/Akt pathway, which critically involves in angiogenesis [143]. Furthermore, production of extracellular vesicles (EVs), such as exosomes, is also implicated in the mediation of ethanol-induced vascularization [144]. The ethanol-upregulated EVs contain long non-coding RNAs HOTAIR and MALAT1 [144]. 

Besides the regulation of angiogenesis by means of enhancement or inhibition of endothelial cell proliferation, exposure to ethanol may affect the cellular phenotype of endothelial cells. In our recent study, the subacute administration of ethanol (2 g/kg b.w. for 4 days) induced abnormal blood vessel structures in the cerebral cortex, which was not accompanied by apparent endothelial cell proliferation (Hasegawa et al., manuscript submitted). In these settings, VEGF-A expression is suppressed after exposure to ethanol. Therefore, we believe that ethanol can induce blood vessel remodeling without affecting angiogenesis.

## 3. Conclusions

Use of ethanol is widespread, and it has been an integral part of human history and culture. However, its detrimental effects on our health and social behavior have rendered ethanol and ethanol-related toxicity as significant issues. From this point of view, understanding the toxic effects of ethanol is an important scientific issue. Recent studies have revealed both direct and indirect biological pathways involved in the neurological effect of ethanol. Especially, research has demonstrated that endothelial cells play a critical role as blood–brain barriers for ethanol penetration into the nervous system and as a center for producing intercellular signaling molecules. Endothelial cells can therefore be a potent target for managing the ethanol toxicity. However, it is important for future studies to address the following issues: (1) the molecular target of ethanol in endothelial cells should be identified; (2) the sensitivity of endothelial cells against ethanol, and the signaling pathway in endothelial cells exerting an ethanol-induced toxic effect should be controlled; and (3) the downstream biological events from endothelial cells, that is the production and function of angiocrine factors, should be understood and controlled.

## Data Availability

Not applicable.
